# Supplementary material for: Vine Tea Extract (VTE) Inhibits High-Fat Diet-Induced Adiposity: Evidence of VTE’s Anti-Obesity Effects In Vitro and In Vivo
Source: Int J Mol Sci. 2024 Nov 9;25(22):12042. doi: 10.3390/ijms252212042 (PMC11593453; doi:10.3390/ijms252212042)
Supplement: Supplementary file 1 [file ijms-25-12042-s001.zip › ijms-3253628-supplementary.pdf]

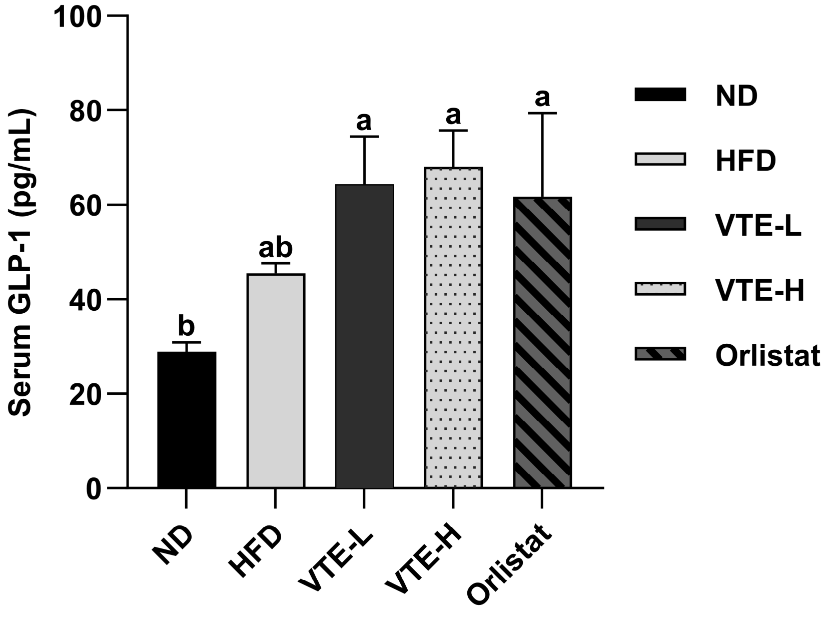

Figure S1. VTE regulates serum GLP-1 in HFD-induced obese mice. The level of GLP-1 in the serum of the mice was measured using an ELISA kit. Statistical analysis was performed using the one-way ANOVA test followed by the Tukey's multiple comparison test. Differential letters (a and b) mean statistically significant differences among the groups ($p < 0.05$).
